# Optimized expression of *Plasmodium falciparum *erythrocyte membrane protein 1 domains in *Escherichia coli*

**DOI:** 10.1186/1475-2875-3-50

**Published:** 2004-12-15

**Authors:** Kirsten Flick, Sanjay Ahuja, Arnaud Chene, Maria Teresa Bejarano, Qijun Chen

**Affiliations:** 1 Microbiology and Tumor Biology Centre (MTC), Karolinska Institutet, Stockholm, Sweden; 2 Center for Infectious Medicine, Department of Medicine, Karolinska University Hospital, Stockholm, Sweden; 3 Swedish Institute for Infectious Disease Control, Box 280, 171 77, Stockholm, Sweden

## Abstract

**Background:**

The expression of recombinant proteins in *Escherichia coli *is an important and frequently used tool within malaria research, however, this method remains problematic. High A/T versus C/G content and frequent lysine and arginine repeats in the *Plasmodium falciparum *genome are thought to be the main reason for early termination in the mRNA translation process. Therefore, the majority of *P. falciparum *derived recombinant proteins is expressed only as truncated forms or appears as insoluble inclusion bodies within the bacterial cells.

**Methods:**

Several domains of PfEMP1 genes obtained from different *P. falciparum *strains were expressed in *E. coli *as GST-fusion proteins. Expression was carried out under various culture conditions with a main focus on the time point of induction in relation to the bacterial growth stage.

**Results and conclusions:**

When expressed in *E. coli *recombinant proteins derived from *P. falciparum *sequences are often truncated and tend to aggregate what in turn leads to the formation of insoluble inclusion bodies. The analysis of various factors influencing the expression revealed that the time point of induction plays a key role in successful expression of A/T rich sequences into their native conformation. Contrary to recommended procedures, initiation of expression at post-log instead of mid-log growth phase generated significantly increased amounts of soluble protein of a high quality. Furthermore, these proteins were shown to be functionally active. Other factors such as temperature, pH, bacterial proteases or the codon optimization for *E. coli *had little or no effect on the quality of the recombinant protein, nevertheless, optimizing these factors might be beneficial for each individual construct. In conclusion, changing the timepoint of induction and conducting expression at the post-log stage where the bacteria have entered a decelerated growth phase, greatly facilitates and improves the expression of sequences containing rare codons.

## Background

Qualitative and quantitative production of proteins in heterologous systems is essential for the characterization of any molecule, from determination of antigenicity, functional and structural analysis to vaccine development. Malaria antigens are among the most difficult proteins to express with *in vitro *methods because of their extreme genetic codon usage. Different organisms have been applied for the production of malaria proteins, including *Escherichia coli *[1,2], baculovirus [3,4], yeast (*Pichia pastoris *and *Saccharomyces cerevisiae*) [5-8], transgenic tobacco plants [9] and transgenic mice [10]. Among these, the *E. coli *expression system is the most attractive and most frequently used, because it quickly produces large amounts of biomass without sophisticated laboratory equipment and at low costs. However, the quality of many proteins expressed in *E. coli *has not been satisfactory. In many cases, the recombinant proteins are either expressed as truncated forms or precipitate in insoluble inclusion bodies in the bacterial cells. Although methods have been developed to obtain correctly folded proteins from these inclusion bodies, the process of refolding cannot be successfully applied to all proteins [11,12].

Proteins expressed in insect cells using the baculovirus system are generally correctly folded [4]. However, so far only a few proteins have been successfully produced using this system because many proteins turned out to be toxic to the insect cells. In addition, the system achieves limited yields, which makes large-scale production cost ineffective. In recent years, expression of malaria proteins in yeast cells including *P. pastoris and S. cerevisiae *has been established in several laboratories [5-8]. Recombinant CSP, MSP-119, MSP-1-AMA-1 hybrid proteins and the cysteine-rich inter-domain region (CIDR) of a *Plasmodium falciparum *erythrocyte membrane protein 1 (PfEMP1) have been produced in *P. pastoris *for malaria vaccine studies in either primates or pre-clinical trials in humans [13]. However, for expression in *P. pastoris*, the codon sequences of these antigens need to be optimized. In most cases, sequences encoding for the amino acids of potential glycosylation sites have to be removed. So far, this system is the most promising one and might be the favourite choice when it comes to the production of recombinant malaria proteins under GMP conditions. It is nevertheless unlikely that this system will replace *E. coli *as a routine bench bioreactor due to its complicated manipulation and relatively long cultivation times. The use of long synthetic peptides (LSP) has been explored in malaria vaccine antigen production in recent years [14,15]. The advancing technology of peptide biosynthesis has made it possible to produce LSP with a high degree of homogeneity and purity. Furthermore, LSP can be designed in a way that they contain a large number of T cell epitopes, which leads to the generation of stronger CTL-mediated immune responses. However, this technology also has its limitations: it is still difficult to manufacture peptides that are more than 100 amino acids in length and proteins with multiple disulfide bonds are generally complicated to produce.

The *P. falciparum *genome is one of the most A/T-rich genomes. Surface exposed molecules expressed by the parasite such as members of the PfEMP1 family are positively charged, caused by the abundance of arginine and lysine residues in their sequences, which complicates their expression in heterologous systems such as *E. coli*. The high content of A/T repeats in the mRNA template is a reason for early translation termination and results in heterogeneity of recombinant proteins. Members of the PfEMP1 family are of great interest since they are virulence factors that mediate adhesion of *P. falciparum*-infected erythrocytes in the post-capillary microvasculature, a process that leads to severe malaria [16]. Each PfEMP1 molecule is composed of several Duffy-binding like domains (DBL) and CIDR domains. Both DBL and CIDR domains have a distinct number of cysteine residues and several lysine and arginine motifs [17]. Molecular characterization, including antigenic analysis of this protein family, relies in most cases on the successful *in vitro *production of the correctly folded protein, and production of these proteins in the native conformation remains particularly difficult.

In most studies where *E. coli *was used as a bioreactor, it has been the main goal to reach as high expression levels of the recombinant protein as possible. However, high expression levels will not guarantee a high quality of the final product. Efficient expression of heterologous proteins in *E. coli *is impaired by the rarity of certain tRNAs that are abundant in the organisms from which the heterologous protein is derived [1]. When the process of expression achieves high levels, the limited amounts of the tRNAs will quickly be exhausted. The lack of tRNAs will result in a drop-off of the ribosomal unit from the mRNA template and will terminate the translation process.

This study describes a simple method for optimizing the cultivation conditions and especially the timepoint of induction exemplified by the expression of several PfEMP1 domains in *E. coli*. Induction at the post-log stage of bacterial growth leads to the production of considerably larger amounts of soluble protein of a higher quality compared to standard conditions. Moreover, the method is easily applicable in laboratories where a sophisticated cultivation facility is not available.

## Methods

### Parasites

The *P.falciparum *parasite strains FCR3S1.2 and TM284S2 were cultured according to standard methods with 10% AB^+ ^Rh^+ ^serum added to the buffered medium (RPMI supplemented with Hepes, gentamycin and sodium bicarbonate). Genomic DNA from these parasites was purified using the EasyDNA purification kit (Invitrogen) according to the manufacturer's protocol.

#### Recombinant plasmids

Plasmid constructs for the expression of the recombinant proteins GST-DBL1α and GST-CIDR1α of FCR3S1.2*var*1PfEMP1, GST-DBL1α and GST-DBL2β of TM284S2*var*1PfEMP1 were generated as described earlier [18,19].

#### Codon optimization and gene resynthesis

The sequence of the DBL1α domain of FCR3S1.2*var*1PfEMP1 was optimized for codon adaptation in *E. coli*. The genes were re-synthesized chemically (GeneArt, Germany). The re-synthesized DBL1α was amplified with oligonucleotide primers (rDBL-1 5'-ATG GCT ACT TCC GGA GGA, rDBL-1.1 5'-TTC GAT AAG CAG AAG AAG TAC) and cloned into the pGEX4T-1 vector as described [20].

#### E. coli strain

The BL21-CodonPlus-RIL strain purchased from Stratagene (California, USA) was used for protein expression. This bacterial strain has been engineered to contain a high copy number of argenine*U*-, leucine*W*-and isoleucine*Y*-tRNA genes for optimal expression of heterologous proteins of organisms with A/T-rich genetic sequences.

#### Expression of PfEMP1 domains in BL21-CodonPlus-RIL bacteria

BL21 competent cells were transformed with recombinant pGEX4T-1 plasmids containing FCR3S1.2 DBL1α, TM284S2 DBL1α or TM284S2 DBL2β as inserts. The transformed bacteria were selected on LB agar plates containing ampicillin (100 μg/ml). A single colony of the transformed bacteria was inoculated in 30 ml LB medium containing ampicillin (100 μg/ml) and chloramophenicol (50 μg/ml) for cultivation at 37°C overnight. Aliquots of the culture were inoculated into one litre LB medium with ampicillin (100 μg/ml). The cultivation was carried out with a shaking speed of 225 rpm. The pH value and the optical density at A_600 _of the cultures were monitored systematically. Aliquots (50 ml) of each culture were sequentially taken after the OD A_600 _reached 0.5 and IPTG (isoprophyl-b-D-thiogalactopyranoside) was added to a final concentration of 0.1 mM to induce the expression. The expression was carried out for three hours at 37°C and the bacteria were harvested afterwards by centrifugation at 4000 rpm for 15 minutes. The recombinant proteins were purified on Glutathione-sepharose (Amersham-Phamacia, Sweden) as described earlier [18,19].

#### SDS-PAGE analysis of the recombinant proteins

To analyse the recombinant proteins, aliquots of the soluble and insoluble fractions of the expressed proteins from each purification were mixed with an equal volume of SDS-PAGE loading buffer containing β-mercaptoethanol and boiled at 100°C for 5 min. The denatured proteins were resolved in 10% acrylamide gels containing 1% SDS and visualized by staining in Coomassie brilliant blue solution.

#### Binding to heparin and blood group A antigen

Purified recombinant DBL1α of FCR3S1.2 expressed with pGEX plasmids containing either the wild-type DBL1 sequence or the codon-optimized sequence was further passed through a heparin-HiTrap column (Amersham-Phamacia Biotech, Sweden). After washing with PBS tween-20 buffer, the bound protein was released from the column with 2M NaCl and dialyzed immediately against cold PBS. Aliquots of the eluted proteins were subjected to SDS-PAGE.

The binding of recombinant DBL1α of FCR3S1.2 to blood group A antigen was studied using a solid phase assay system as described earlier [20].

## Results and discussion

**Figure 1:**
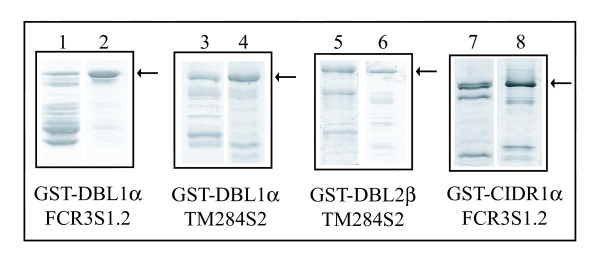
Comparison of recombinant DBL, CIDR proteins expressed at mid-or post-log phase. Expression of GST-DBL1α and GST-CIDR1α of FCR3S1.2, GST-DBL1α and GST-DBL2β of TM284S2 was induced when the bacterial growth was at mid-log respectively post-log stages. The purified recombinant proteins were analysed in SDS-PAGE. Results presented in lane 1, 3, 5 and 7 are proteins purified from cultures where expressions was initiated at mid-log phase, while lane 2, 4, 6 and 8 show proteins after induction at post-log phase. The intact fraction of each expressed protein is marked with an arrow. Proteins were in addition verified by Western-Blot both with anti-GST-and anti-DBL1-antibodies [26].

The expression of three different DBL-domains and one CIDR-domain as recombinant proteins in *E. coli *was induced either at an OD A_600 _of 0.6, which is commonly recommended or at an OD A_600 _higher than 2.0. SDS-PAGE analysis (Figure 1) shows that most of the recombinant proteins of the cultures induced at a low OD A_600 _(Figure 1, lane 1, 3, 5, 7) were truncated at the C-terminal end displaying multiple bands of different molecular weights, while the intact protein represents only a small fraction of the overall protein yield. In contrast, if the expression was induced at a higher OD A_600 _(Figure 1, lane 2, 4, 6, 8), the dominant fraction of the protein was found to be the intact form, which proved to be true for all four domains tested although derived from different PfEMP1s.

In bacterial cultures, the growth will be at log phase between an OD A_600 _of 0.3 and 1.5. During the log phase, the number of bacteria in the culture doubles approximately every 20 minutes. Afterwards, the proliferation rate slows down due to the lack of nutrients. If the induction is initiated while the bacteria grow in log phase, the bacterial translation machinery will be highly active and the expression of the recombinant protein follows this profile, because once turned on, the promoter controlling the heterologous sequence on the vector does not underlie further control mechanisms. During expression, the rare codons of arginine, leucine, isoleucine and proline frequently found in PfEMP1 sequences will inhibit the translation process, most likely caused by the exhaustion of the tRNAs for these amino acids. It has been reported that the rare codons of arginine and proline are likely to cause frameshifts and with that undesired products in bacterial expression system [21-23]. The data reported here indicate that these problems mainly occur during the high-level expression stage, since proteins expressed at post-log growth stage are much less truncated.

Enzymatic digestion of heterologous proteins in *E. coli *is thought to be an additional reason for product heterogeneity of recombinant proteins [24]. The experiments of this study could not confirm degradation by bacterial proteases as one of the major causes, since the use of a protease inhibitor cocktail in the purification protocol did not affect the pattern of the expressed products (data not shown). In addition, expression was carried out using a BL21 Codon Plus bacterial strain (Stratagene) that is deficient in the OmpT and Lon bacterial proteases.

**Figure 2:**
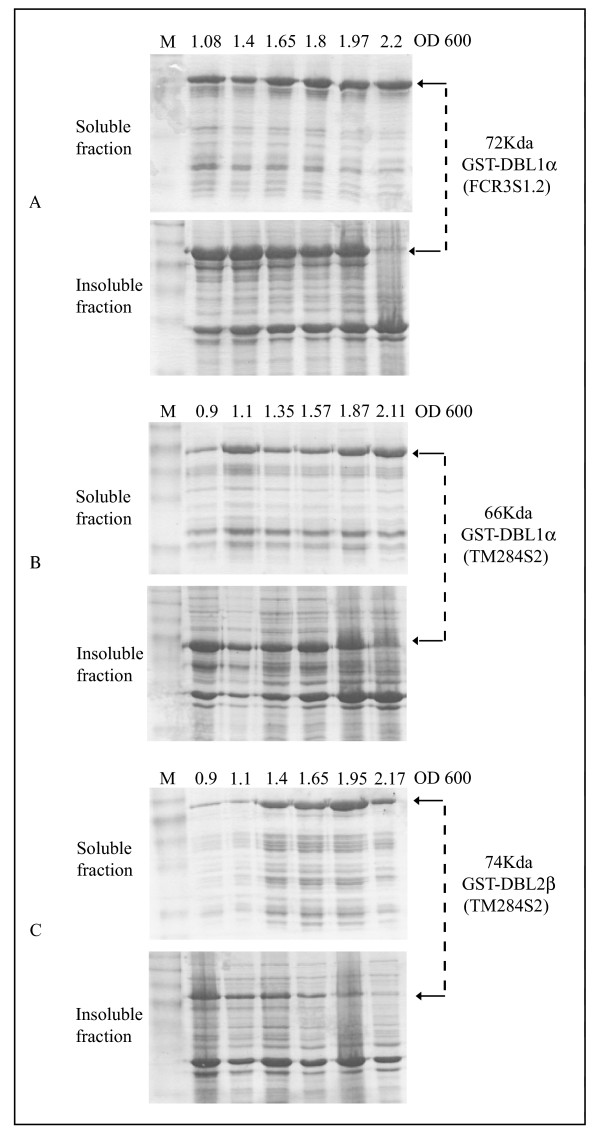
Impact of bacterial growth stages on protein solubility. The expression of the three recombinant proteins was induced at various timepoints chosen gradually from low OD A_600 _to higher OD A_600_. The comparison of the soluble and insoluble fractions of the recombinant proteins revealed that after initiation of expression at an OD A_600 _of greater than 2.0, the recombinant proteins are found almost completely in the soluble fraction. The amount of each protein loaded is not proportional to the size of the cultivation.

We have previously found that a large proportion of the recombinant proteins remain in the insoluble fraction whereas only small amounts appear in the soluble fraction (Figure 2 and data not shown) if expression is initiated at an OD A_600 _of 0.6. To check whether the bacterial growth status at the induction timepoint has any effect on protein solubility, induction of expression was carried out on aliquots of the same bacterial stock culture at different bacterial densities (OD A_600 _value). Both soluble and insoluble fractions of the same culture were compared. The results (Figure 2A–C) clearly show that the majority of the three recombinant proteins remain in the insoluble fraction when the expression was induced at an OD A_600 _below 2.0. If, on the other hand, the induction is initiated at an OD A_600 _greater than 2.0, almost the total amount of the recombinant proteins appears in the soluble fraction.

The solubility of a protein correlates with its correct structure that is formed during a post-translational folding process. Freshly synthesized polypeptides remain in a stage of intermediate form in the bacterial cytoplasma. After several enzymatic and biochemical processing steps, the peptides are folded into their functional form [24]. However, if proteins are folded incorrectly, they tend to accumulate as aggregates in the bacterial cell and, in order to avoid toxic effects on the host system, the bacteria store these aggregates in confined structures referred to as inclusion bodies.

Formation and accumulation of heterologous proteins as inclusion bodies is a common problem in protein expression. The exact mechanism of this process is still not understood. It has been suggested that factors such as culture pH, temperature and protein amino acid composition might affect the solubility of a recombinant protein [24]. The data reported here indicate that the expression speed and, with that, the subsequent folding process is the most important factor. Protein expression at the post-log phase resulted in high amounts of soluble protein, which indicates that at this stage the low bacterial growth rate implicates a biosynthesis process that is kept at low speed. The slow synthesis process will allow the protein processing machinery to efficiently assemble the freshly synthesized peptides into the correct structure. Correctly folded proteins are most likely to stay in the soluble form provided that the molecule does not contain large numbers of hydrophobic residues.

Although we found that the pH value of the growing culture is influenced by the amino acid composition of the expressed polypeptide, keeping a stable pH value in the bacterial culture does not affect the protein solubility (data not shown) and, therefore, has little influence on the quality of the expressed protein. However, temperature is an important factor to consider. Keeping the culture at 16°C before and after induction slightly improves the protein quality (data not shown), but, on the other hand, slows down bacterial growth considerably and therefore minimizes the final yield of the recombinant protein.

It has been reported that codon-optimized sequences for the use in *E. coli *will improve expression quality. Here we show that C/G versus A/T contents of the heterologous gene sequence are not among the most important factors that determine the quality of the recombinant protein. The expression of GST-DBL1α of FCR3S1.3 (Figure 2A) optimized for expression in *E. coli *shows a very similar expression pattern compared to those ones of GST-DBL1α and GST-DBL2β of TM284S2 which were expressed using the wildtype *P. falciparum *sequences (Figure 2B,C). This indicates that sequence composition is not always a determinant factor for expression quality.

**Figure 3:**
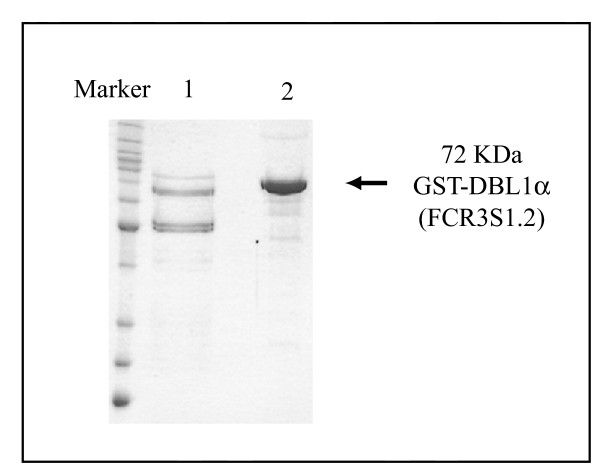
Binding to heparin. Recombinant GST-DBL1α of FCR3S1.2 was purified from cultures with induction at mid-log (lane 1) and post-log stage (lane 2) and bound to heparin. Protein expressed by bacteria at post-log stage showed considerably higher affinity to heparin.

We have previously found that the DBL1α domain of FCR3S1.2var1 PfEMP1 binds to the human erythrocyte surface through heparan sulfate [20,25]. Further, the recombinant GST-DBL1α of FCR3S1.2 protein can be purified through binding to heparin-sepharose. In this study, the same amount of GST-DBL1α purified from cultures of expression started at an OD A_600 _of 0.6 and greater than 2.0 was tested for its ability to bind to heparin. Although the truncated forms of the DBL1 display binding to heparin due to the presence of heparin-binding motifs in these peptides, there is a remarkable difference in terms of binding affinity between the proteins expressed at different bacterial densities as shown in Figure 3. Proteins expressed at a high OD A_600 _are not only more intact and more soluble, but also display higher affinity to heparin.

To further demonstrate functionality of the proteins expressed at high OD A_600 _the DBL1α of FCR3S1.2 was subjected to a blood group A binding assay, which confirmed the specific interaction between the DBL1α and the blood group A antigen (data not shown).

The expression of *P. falciparum *derived proteins, especially membrane-bound proteins is still a great challenge due to the high content of amino acids encoded by rare codons in the *P. falciparum *genome. The method reported here presents an easily applicable tool to express sequences containing rare codons. The key factor for the expression of such proteins is to decelerate the translation machinery inside the bacteria. Low expression speed will not only allow the ribosomal unit to smoothly pass through the mRNA templates and synthesize full-length polypeptide chains, but also enable the proteins to slowly transfer from the unstable intermediate phase to the correctly folded phase. The described expression approach will result in a final product that is soluble, intact and functional, nevertheless, additional factors might influence the expression and need to be optimized for each individual construct.

The expression of eukaryotic genes in *E. coli *is one of the most frequently used tools in modern science. Numerous approaches have aimed at achieving the highest possible level of expression by having a maximum amount of protein expressed per bacterial cell. Our studies suggest on the contrary that increasing the number of bacterial cells in the culture while at the same time keeping the expression process at a low profile, might considerably improve the quality and quantity of the protein. That way, high level expression can simply be achieved by increasing the bacterial density of a culture, whereby problems in form of truncated or insoluble protein factions are almost completely eliminated.

## Authors' contributions

KF carried out the expression assays. SA and AC participated in the expression and optimization experiments. MTB participated in sequence design. QC coordinated the experiments and helped to draft the manuscript. All authors read and approved the final manuscript
